# Supplementary material for: Zebrafish Model as a Screen to Prevent Cyst Inflation in Autosomal Dominant Polycystic Kidney Disease
Source: Int J Mol Sci. 2021 Aug 20;22(16):9013. doi: 10.3390/ijms22169013 (PMC8396643; doi:10.3390/ijms22169013)
Supplement: Supplementary file 1 [file ijms-22-09013-s001.zip › ijms-1286974-supplementary.pdf]

Supplementary Material:

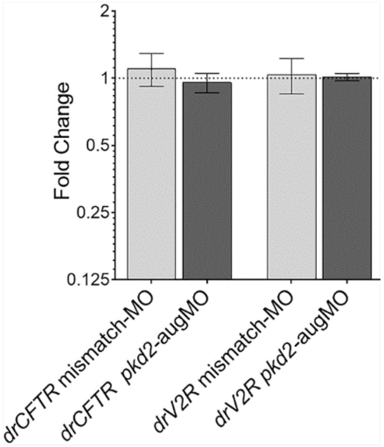

Figure S1 - mRNA levels of V2R and CFTR. Normalised fold expression (Log2) of the two genes are presented as determined in a KV specific microarray data (unpublished data) for both mismatch-MO control (light grey bars) and *pkd2*-knockdown embryos (dark grey bars).

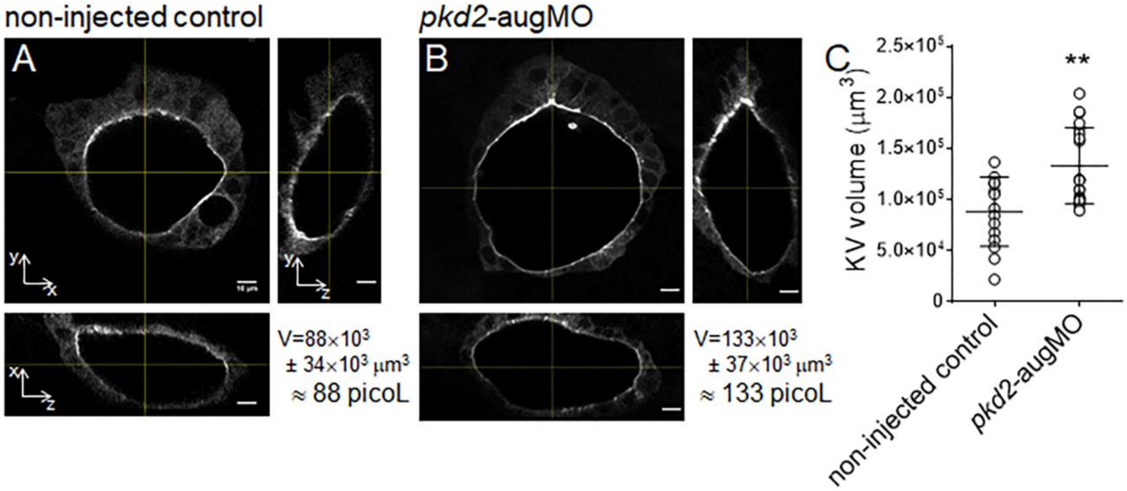

Figure S2 – *TgBAC(cftr-GFP)pd1041* embryos KV Volume. (**A, B**) Confocal live-microscopy scans of the whole KV of 10-11 ss *TgBAC(cftr-GFP)pd1041* embryos. The middle focal plane along the *xy* axis and the respective orthogonal views (along *xz* and *yz* axes) are shown for the most representative (A) non-injected control and (B) *pkd2*-knockdown embryos. KV_{volume} is indicated in μm^3 and picoL. (**C**) Estimated KV volumes (μm^3) for non-injected control (n=15) and *pkd2*-knockdown (n=20) embryos. Mean±SD are indicated. As the pkd2-augMO injected embryos sample did not follow a normal distribution, the Mann-Whitney test was used to compare the *pkd2*-augMO *versus* non-injected embryos, $^{**}p<0.01$. Scale bars: 10 μm.
